# The Association between Infections and General Cognitive Ability in Young Men – A Nationwide Study

**DOI:** 10.1371/journal.pone.0124005

**Published:** 2015-05-13

**Authors:** Michael Eriksen Benros, Holger Jelling Sørensen, Philip Rising Nielsen, Merete Nordentoft, Preben Bo Mortensen, Liselotte Petersen

**Affiliations:** 1 Mental Health Centre Copenhagen, University of Copenhagen, Faculty of Health Sciences, Bispebjerg Bakke 23, 2400 Copenhagen NV, Denmark; 2 National Centre for Register-based Research, Aarhus University, Fuglesangs allé 4, 8210 Aarhus V, Denmark; 3 The Lundbeck Foundation Initiative for Integrative Psychiatric Research, *i*PSYCH, Aarhus, Denmark; University of Pécs Medical School, HUNGARY

## Abstract

**Background:**

Infections and activated immune responses can affect the brain through several pathways that might also affect cognition. However, no large-scale study has previously investigated the effect of infections on the general cognitive ability in the general population.

**Methods:**

Danish nationwide registers were linked to establish a cohort of all 161,696 male conscripts during the years 2006–2012 who were tested for cognitive ability, which was based on logical, verbal, numerical and spatial reasoning at a mean age of 19.4 years. Test scores were converted to a mean of 100.00 and with a standard deviation (SD) of 15. Data were analyzed as a cohort study with severe infections requiring hospitalization as exposure using linear regression.

**Results:**

Adjusted effect sizes were calculated with non-exposure to severe infections as reference, ranging from 0.12 SD to 0.63 SD on general cognitive ability. A prior infection was associated with significantly lower cognitive ability by a mean of 1.76 (95%CI: -1.92 to -1.61; corresponding to 0.12 SD). The cognitive ability was affected the most by the temporal proximity of the last infection (P<0.001) and by the severity of infection measured by days of admission (P<0.001). The number of infections were associated with decreased cognitive ability in a dose-response relationship, and highest mean differences were found for ≥10 hospital contacts for infections (Mean: -5.54; 95%CI: -7.20 to -3.89; corresponding to 0.37 SD), and for ≥5 different types of infections (Mean: -9.44; 95%CI: -13.2 to -5.69; corresponding to 0.63 SD). Hospital contacts with infections had occurred in 35% of the individuals prior to conscription.

**Conclusions:**

Independent of a wide range of possible confounders, significant associations between infections and cognitive ability were observed. Infections or related immune responses might directly affect the cognitive ability; however, associated heritable and environmental factors might also account for the lowered cognitive ability.

## Introduction

It is increasingly recognized that infections and immune responses can affect the brain and activate immunocompetent cells within the brain [1–3], influencing on neuronal signal transduction and possibly cognition [4–6]. Impaired cognition has been observed in association with elevated levels of CRP and several infections, such as herpes virus, hepatitis and HIV in smaller studies [7–9]. Furthermore, experimental activation of inflammatory reactions in healthy volunteers has been shown to induce reduced cognitive performance [10]. Particularly patients with sepsis and encephalitis have been shown to have affected cognition in long time periods after the infection has been cleared, thus infections might also have a longer lasting effect on cognition [2,11]. In old individuals, especially those with dementia, infections are considered the main triggers of delirium [11], which is also associated with long-term cognitive impairment [12]. Infections and immune responses have additionally been associated with several mental disorders with affected cognition, such as schizophrenia and mood disorders [13–15]. Furthermore, experimental induction of infections and inflammation has in animal studies been shown to affect the brain and induce neuropsychiatric symptoms [14]. Nonetheless, large-scale longitudinal studies are lacking on the association between infections and cognitive ability in the general population.

We utilized nationwide draft board tests on general cognitive ability in male conscripts in Denmark, to explore the relationship between infections—from birth up to adolescence—on cognitive ability around age 19 years. In an effort to avoid or reduce the risk of bias due to confounding factors, we examined whether an independent effect of infections on general cognitive ability could be detected in a model adjusting for other factors known to influence the cognitive ability (e.g. parental education, gestational age, birth weight and birth order) [16]. Additionally, we explored a possible dose-response relationship, the temporal associations, severity, and the effect of the type and site of the infection.

## Materials and Methods

### Study population

This study was based on cognitive ability test data from the Danish draft board collected in the Danish Conscription Registry [17], a nationwide register including approximately 190,000 men born in Denmark between 1976 and 1994 assessed between 2006 and 2012. Denmark maintains military conscription for which all men become liable at age 18; however, men with conditions such as severe mental retardation, asthma and extreme myopia are exempted from conscription (approximately 10–15%), but not all mental health problems are regarded as disqualifiers for military service [18,19]. All Danish residents are assigned a unique personal identifier in all Danish national registers, enabling unique linkage between registers. All individuals were followed in the Danish National Hospital Register for the occurrence of hospital contacts with infections from 1977–2012 [20]. From the Danish Civil Registration System information on parental age, birth order and twin status were obtained [21]. The Danish Medical Birth Registry contains maternal and obstetric information, such as gestational age and birth weight [22]. Information on parental education was retrieved from Statistics Denmark’s database IDA [23]. Information about psychiatric diagnoses was obtained from the Danish Psychiatric Central Research Register [24]. We excluded 10,579 conscripts born abroad and 21,582 with parents not born in Denmark, accordingly the analyses included 161,969 conscripts. The described nationwide Danish registers are available to all researchers in Denmark after approval from the Danish Data Protection Agency.

### Exposure measure: Assessment of infections

A history of infection was defined as the person having been recorded with a diagnosis of infection in the Danish National Hospital Register. We omitted all diagnoses that bore the modification code “suspected” or “not found”. The diagnostic codes and grouping according to type and site of the infections is described in the S1 Table.

### Outcome measure: Assessment of cognition measured from draft board data

The Børge Prien’s Prøve (BPP) is a Danish intelligence test that has been used by the Danish Draft Board examination since 1956 and is a test for cognitive ability [18]. The test consists of 4 subtests each with about 20 items (78 in total), designed to assess logical, verbal, numerical and spatial reasoning. The tests are timed (45 minutes) and the result is number of correct answers to the 78 questions, but this cognitive ability score has here been converted to parallel conventional IQ scaling, with a mean of 100 and an SD of 15, to give a more familiar metric of effect size, which has been used in previous publications [25]. The test has satisfactory test-retest reliability of 0.77 [26], and correlates with educational achievement and with the Wechsler Adult Intelligence Scale (correlation = 0.82) [27].

### Statistical analysis

We estimated the mean differences in cognitive ability scores and likelihood ratio based 95% confidence intervals according to hospital contacts with infections using linear regression in Stata 12 (Stata, College Station, Texas, United States). Mean values of the BPP test have changed over recent decades, thus it is recommended that studies account for year of testing [18], and all estimates in the basic adjustment model were adjusted for year of testing. Estimates in the fully adjusted model were all additionally adjusted for the following variables that had previously been associated with cognitive ability and used in prior publications [16,28]: paternal and maternal education divided to 5 levels (measured when the conscript was around 18 years of age), birth order (first, second, third and fourth or later born, multiple birth status (singleton versus twin/multiple), birth weight and gestational age (defined as the lowest 10% for a given gestational week), and lastly also for an individual or parental history of psychiatric disorders and substance abuse. Conscripts with the same mother (i.e. brothers or maternal half-brothers) comprised a cluster, and allowance for possible within-cluster dependence was made by using robust standard error estimates provided by the cluster option in Stata. To distinguish effects of restricting to term babies (that is, gestational age of 37 weeks or later) and the effect of adjustment, an additional set of sensitivity analyses were conducted on initial models in term babies (week 37–42, singletons and birth weight 3–4 kilo). We also included parental infections in the additional analysis. Furthermore, we conducted sensitivity analysis excluding individuals with a cancer diagnosis before conscription to evaluate the effect of a severe somatic disease on the associations (ICD-8: 140–209 and ICD-10: DC00-96 in the Danish National Hospital Register)

## Results

The cohort consisted of all 161,696 male conscripts born in Denmark and with Danish parents that were tested for cognitive ability during the years 2006–2012. Out of these, 93,504 were born at term. The mean age of the 161,696 conscripts at testing was 19.4 years. The overall mean of the cognitive scores was 100.0 and with a standard deviation of 15.0 units after conversion to parallel conventional IQ scaling (higher scores indicate better cognitive ability). A total of 56,258 individuals had a prior hospital contact for infections, equaling 34.79% of the individuals tested for cognitive ability.

We found that any hospital contact with infection was associated with a mean score of 1.76 units lower cognitive ability (95%CI: –1.92 to -1.61; corresponding to 0.12 SD), when compared to people with no hospital contacts for infections (Table 1). In the full adjustment model, the association between infections and lower cognitive ability persisted (mean: -1.13; 95%CI: –1.27 to -0.98). When looking at the localization of the infection, CNS infection was associated with the largest decline in cognitive ability (mean -2.38; 95%CI: –2.70 to -2.06) as also depicted in Table 1.

**Table 1:** The effect on cognition of a hospital contact among persons with infections according to the infection site.^1^

		Basic adjustments^2^	Fully adjusted^3^
	N	Mean diff. 95% CI	Mean diff. 95% CI
**Infection during childhood and adolescence**			
Yes	56,258	**-1.76** (-1.92 to -1.61)	**-1.13** (-1.27 to -0.98)
No	105,438	0.00 (ref)	0.00 (ref)
**Type of infection** ^4^			
Any viral	17636	**-1.34** (-1.56 to -1.13)	**-0.82** (-1.02 to -0.62)
Any bacterial	22266	**-1.55** (-1.78 to -1.31)	**-1.05** (-1.27 to -0.83)
Any other type	32943	**-2.10** (-2.28 to -1.91)	**-1.27** (-1.44 to -1.10)
Persons without a hospital contact with infection (reference)	105438	0.00 (ref)	0.00 (ref)
**Site of infection** ^5^			
CNS infections	9585	**-2.38** (-2.70 to -2.06)	**-1.39** (-1.69 to -1.10)
Sepsis infections	983	**-1.60** (-2.60 to -0.60)	-0.75 (-1.68 to 0.17)
Otitis media infection	1078	**-1.79** (-2.69 to -0.88)	**-1.00** (-1.84 to -0.16)
Gastrointestinal infections	9456	**-1.73** (-2.04 to -1.42)	**-0.99** (-1.28 to -0.69)
Skin infection	7705	**-1.58** (-1.93 to -1.23)	**-1.16** (-1.48 to -0.84)
Respiratory infections	26445	**-1.69** (-1.89 to -1.49)	**-1.00** (-1.19 to -0.81)
Urological infections	1571	**-1.29** (-2.06 to -0.52)	**-0.90** (-1.60 to -0.20)
Genital infection	6	-	-
Hepatitis infections	56	-1.35 (-5.55 to 2.85)	-0.79 (-4.66 to 3.09)
Other types of infections	20462	**-1.84** (-2.06 to -1.61)	**-1.20** (-1.40 to -0.99)
Persons without a hospital contact with infection (reference)	105438	0.00 (ref)	0.00 (ref)

^1^ The cognitive ability score has been converted to parallel conventional IQ scaling, with a mean of 100 and an SD of 15, to give a more familiar metric of effect size. Boldface indicates that the 95% confidence interval did not include 0.0.

^2^ Adjusted for year of testing

^3^ Additionally adjusting for paternal and maternal education, measured when the conscript was around 18 years of age, birth order, multiple birth status, birth weight and gestational age, and lastly also for individual and parental history of psychiatric disorders and substance abuse

^4^ Calculated in 3 separate analyses. Notice that one individual may have more than one diagnosis.

^5^ Calculated in 9 separate analyses. Notice that one individual may have more than one diagnosis.

A bacterial infection was associated with a mean score of 1.55 units lower cognitive ability (95%CI: –1.78 to -1.31) and a viral infection was associated with a mean score of 1.34 units lower cognitive ability (95%CI: –1.56 to -1.13). Any other type of infections was associated with a mean score of 2.10 units lower cognitive ability (95%CI: –2.28 to -1.91).

**Fig 1:**
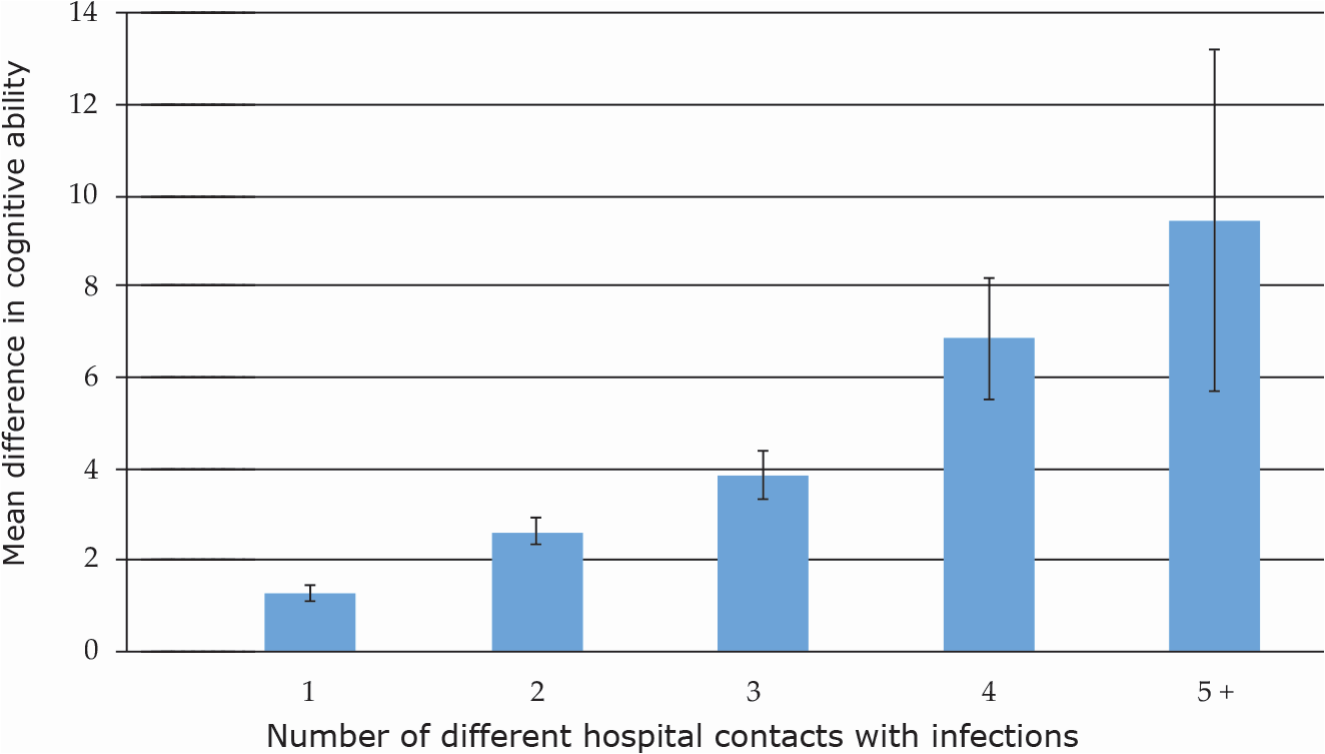
Mean differences, with confidence intervals, of cognitive ability in persons with hospital contacts for infections compared to people with no hospital contacts for infections.

The number of hospital contacts with infection affected the cognitive ability in a dose-response relationship, where the group with 10 or more hospital contacts with infections were associated with a mean score of 5.54 units lower cognitive ability (95%CI: –7.20 to -3.89; corresponding to 0.37 SD; n = 351), compared to people without hospital contacts for infections (Table 2). The associations were greater when looking at individuals with hospital contacts for different types of infections, where individuals with 4 hospital contacts with different types of infections had a mean score of 6.84 units lower cognitive ability (95%CI: –8.17 to -5.50; n = 579) and individuals with 5 or more different types of infections, had a mean score of 9.44 units lower cognitive ability (95%CI: –13.20 to -5.69; corresponding to 0.63 SD; n = 85) (Fig 1). The dose-response relationship was also significant in the fully adjusted model (P<0.001).

**Table 2:** The effect on cognition associated with the number of hospital contacts with infections in Denmark between birth and draft board examination.^1^

	**Number of hospital contacts with infections**		
	**Basic adjustments** ^2^		**Fully adjusted** ^3^
	**N**	**Mean diff. (95% CI)**	**Mean diff. (95% CI)**
Persons without a hospital contact with an infection (ref)	105,438	0.00 (ref)	0.00 (ref)
1 hospital contact with infection	29,881	**-1.16** (-1.36 to -0.97)	**-0.77** (-0.95 to -0.59)
2–4 hospital contacts with infections	22,686	**-2.17** (-2.39 to -1.96)	**-1.41** (-1.61 to -1.21)
5–9 hospital contacts with infections	3,340	**-3.96** (-4.49 to -3.42)	**-2.30** (-2.78 to -1.81)
10 or more hospital contacts with infections	351	**-5.54** (-7.20 to -3.89)	**-2.70** (-4.16 to -1.24)
	**Number of hospital contacts with different sites of infections**		
		**Basic adjustments**	**Fully adjusted**
	**N**	**Mean diff. 95% CI**	**Mean diff. 95% CI**
Persons without a hospital contact with an infection (ref)	105,438	0.00 (ref)	0.00 (ref)
1 hospital contact with infection	39,774	**-1.24** (-1.42 to -1.07)	**-0.83** (-0.99 to -0.67)
2 hospital contacts with infections	12,640	**-2.60** (-2.88 to -2.32)	**-1.64** (-1.89 to -1.37)
3 hospital contacts with infections	3,180	**-3.83** (-4.37 to -3.30)	**-2.18** (-2.68 to -1.69)
4 hospital contacts with infections	579	**-6.84** (-8.17 to -5.50)	**-4.51** (-5.74 to -3.27)
5 or 6 hospital contacts with infections	85	**-9.44** (-13.20 to -5.69)	**-5.29** (-8.84 to -1.74)

^1^ The cognitive ability score has been converted to parallel conventional IQ scaling, with a mean of 100 and an SD of 15, to give a more familiar metric of effect size. Boldface indicates that the 95% confidence interval did not include 0.0.

^2^ Adjusted for year of testing

^3^ Additionally adjusting for paternal and maternal education, measured when the conscript was around 18 years of age, birth order, multiple birth status, birth weight and gestational age, and lastly also for individual and parental history of psychiatric disorders and substance abuse

The effect on cognitive ability increased with the temporal proximity of the last infection (P<0.001) as displayed in Table 3, where a hospital contact for infection within the last year was associated with a mean score of 2.95 units lower cognitive ability (95%CI: -3.52 to -2.38). However, the effect remained significant even if the last infection had occurred in the time period more than 15 years before the cognitive test, which was associated with a 1.62 units lower cognitive ability (95%CI: -1.82 to -1.42), compared to people without hospital contacts for infections.

**Table 3:** The effect on cognitions among persons with infections according to the time since last hospital contact with infection in Denmark (1986–2011).^1^

	Basic adjustments^2^		Full adjustments^3^	
Time since last admission with infection	N	Mean diff. (95%CI)		Mean diff. 95%CI
<1 years	2,615	**-2.95** (-3.52 to -2.38)		**-1.99** (-2.53 to -1.45)
1 years	2,321	**-3.23** (-3.86 to -2.60)		**-2.41** (-3.00 to -1.82)
2 years	2,095	**-2.45** (-3.11 to -1.79)		**-1.68** (-2.29 to -1.06)
3 years	1,689	**-2.79** (-3.50 to -2.09)		**-2.22** (-2.89 to -1.55)
4 years	1,470	**-2.27** (-3.06 to -1.49)		**-1.63** (-2.35 to -0.90)
5–9 years	7,091	**-1.42** (-1.78 to -1.05)		**-0.96** (-1.29 to -0.62)
10–14 years	12,247	**-1.42** (-1.71 to -1.13)		**-0.97** (-1.24 to -0.71)
>15 years	26,730	**-1.62** (-1.82 to -1.42)		**-0.90** (-1.09 to -0.72)
Patients without a hospital contact with infection (ref)	105,438	0.00 (ref)		0.00 (ref)

^1^ The cognitive ability score has been converted to parallel conventional IQ scaling, with a mean of 100 and an SD of 15, to give a more familiar metric of effect size. Boldface indicates that the 95% confidence interval did not include 0.0.

^2^ Adjusted for year of testing

^3^ Additionally adjusting for paternal and maternal education, measured when the conscript was around 18 years of age, birth order, multiple birth status, birth weight and gestational age, and lastly also for individual and parental history of psychiatric disorders and substance abuse

The effects of the age at first/any/latest infection, respectively, on cognition did not differ significantly (data not shown), except that the associations were strongest in the age group 15 years or older, during which any hospital contact for infection was associated with a mean score of 2.35 units lower cognitive ability (95%CI: -2.66 to -2.04).

In order to assess the severity of the infection, we looked at outpatient hospital contacts and days of hospital admittance for infections as inpatient (Table 4). Hospital contacts as an outpatient was associated with a 1.30 units lower cognitive ability (95%CI: -1.64 to -0.96), compared to people without hospital contacts for infections. The cognitive ability decreased in a dose-response relationship with the number of days as an inpatient with a hospital contact for infection (P<0.001), where individuals with 30 days or more as an inpatient was associated with a 6.28 units lower cognitive ability (95%CI: -7.18 to -5.38; n = 1,290), compared to people without hospital contacts for infections. The dose-response relationship was also significant in the fully adjusted model (P<0.001).

**Table 4:** The effect on cognition associated with the number of days as inpatient with infections in Denmark between birth and draft board examination.^1^

Number of hospital contacts with infections			
	Basic adjustments^2^		Fully adjusted^3^
	N	Mean diff. (95% CI)	Mean diff. 95% CI
Persons without a hospital contact with an infection (ref)	105,438	0.00 (ref)	0.00 (ref)
Outpatients	8,160	**-1.30** (-1.64 to -0.96)	**-1.23** (-1.55 to -0.92)
Inpatient 1 day	3,966	**-0.83** (-1.30 to -0.35)	**-0.80** (-1.24 to -0.36)
Inpatient 2 days	9,719	**-1.20** (-1.51 to -0.89)	**-0.85** (-1.14 to -0.56)
Inpatient 3–4 days	12,409	**-1.48** (-1.75 to -1.20)	**-0.93** (-1.19 to -0.67)
Inpatient 5–6 days	6,870	**-1.56** (-1.93 to -1.19)	**-0.88** (-1.22 to -0.53)
Inpatient 7–13 days	9,616	**-2.22** (-2.54 to -1.91)	**-1.28** (-1.57 to -0.99)
Inpatient 14–29 days	4,228	**-3.57** (-4.05 to -3.09)	**-1.89** (-2.34 to -1.44)
Inpatient more than 30 days	1,290	**-6.28** (-7.18 to -5.38)	**-3.45** (-4.31 to -2.59)

^1^ The cognitive ability score has been converted to parallel conventional IQ scaling, with a mean of 100 and an SD of 15, to give a more familiar metric of effect size. Boldface indicates that the 95% confidence interval did not include 0.0.

^2^ Adjusted for year of testing

^3^ Additionally adjusting for paternal and maternal education, measured when the conscript was around 18 years of age, birth order, multiple birth status, birth weight and gestational age, and lastly also for individual and parental history of psychiatric disorders and substance abuse

When additionally adjusting for parental infections the association between infections and lower cognitive ability persisted (mean: -1.52; 95%CI: –1.67 to -1.37) and also in the fully adjusted model when including parental infections in the model (mean: -1.13; 95%CI: –1.27 to -0.90). Nevertheless, having a mother that had a hospital contact with infection was associated with a 0.78 units lower cognitive ability (95%CI: -0.94 to -0.63) and having a father that had a hospital contact with infection was associated with a 0.57 units lower cognitive ability (95%CI: -0.73 to -0.41) in the fully and mutually adjusted model. Furthermore, maternal infections during pregnancy were associated with mean scores 2.68 units below the reference category (95%CI: -3.20 to -2.16) when only including basic adjustments; however, in the fully and mutually adjusted model, maternal infections during pregnancy were associated with mean scores 0.94 units below the reference category (95%CI: -1.43 to -0.45), whereas paternal infections during pregnancy in the fully and mutually adjusted model was not significantly different (mean: -0.24; 95%CI: –1.17 to 0.70).

People with psychiatric disorders did not have a larger effect of infections on cognition than people without psychiatric disorders (P = 0.22), neither did individuals with substance abuse diagnosis (P = 0.86). A family history of psychiatric disorder was associated with a significantly lower cognitive ability after a hospital contact with infection in the basic model (P = 0.01) but not in the fully adjusted model (P = 0.58). There was no significant interaction with a family history of substance abuse (P = 0.12). Among individuals exempted from conscription during our study period, prior hospital contacts with infections had occurred in 43.6%.

When restricting the analysis to term born singletons between 3 and 4 kilograms at birth (n = 93,594), individuals with infections still had a significantly lower general cognitive ability (mean: -1.21; 95%CI: -1.21 to—0.83) and the overall pattern of associations was not changed.

Excluding individuals with a prior cancer diagnosis did not change the estimates, and the effect of the number of infections and duration of hospital admittance were still highly significantly associated with a lower cognitive ability (p<0.0001).

## Discussion

In this national cohort study, we found that hospital contacts for infections were associated with lower general cognitive scores at the draft board examination around age 19. A previous hospital contact with infection had occurred in 35% of the individuals. The number of hospital contacts for infections and the severity of the infection were associated with a decrease in cognitive ability in a dose-response relationship. Furthermore, the reduction in cognitive ability was associated with the temporal proximity of the last infection. The associations between infections and the general cognitive ability persisted after adjusting for a wide range of possible confounders, including parental educational level, year of testing, birth order, multiple birth status, birth weight, gestational age, a parental history of infections, parental and individual history of psychiatric disorders and substance abuse.

The observed associations might be due to a biologically-mediated effect of the infection or associated immune responses causing an acute and possibly transient effect on general cognitive ability. Severe infections as measured by hospital days of admission due to infections decreased the cognitive ability in a dose-response relationship, indicating that the degree of immune response caused by infection were associated with decreased cognitive ability. Inflammation and immune components can directly affect the glutamate, serotonin and dopamine systems that are considered central in cognition [29]. Peripheral inflammation can also indirectly affect the neurotransmitters through the kynurenic pathway without passing the blood-brain barrier [30]. Also, many severe infections might be associated with some degree of sepsis affecting the brain but not giving rise to classical encephalitis symptoms which are very rare. Indeed, follow-up studies in young people with a history of septicaemia have previously shown adverse neurodevelopmental outcomes [31–33]. In line with this, CNS infections were associated with the largest decline of general cognitive ability in our study. However, a bacterial infection was only associated with a marginally larger decrease in cognition than viral infection, and one would expect that bacterial infection is associated with a more extensive immune response. Nevertheless, viral infections such as herpes virus might be present for longer periods with cycles of latency and reactivation in nerve cells that could give cognitive dysfunction due to cumulative damage to brain cells.

Despite that the results remained significant after adjustment for a wide range of confounders, it is appropriate to ask whether individuals exposed to infections differ from those without this exposure in ways relevant to general cognitive ability. It would have been ideal to have some measure of parental general cognitive ability or intelligence, however, the fact that the effect of infection on general cognitive ability was significant after adjustment for parental education may be interpreted as indirect evidence that parental intelligence is not a major confounder of the observed association.

The finding that infections are associated with decreased general cognitive ability after a latency period might be an epiphenomenon and not a biologically-mediated relationship. For instance, a reverse association could have existed so that lower cognitive ability may be a risk factor for acquiring infections. Furthermore, studies have indicated that immune related genes might be implicated in cognition [34], and individuals with genetic liability towards a lower general cognitive ability might also be more genetically vulnerable towards infections. However, although a genetic or reverse association could have partly explained some of the observed associations, this probably does not account for the observed temporal associations or the associations with severity of the infection.

The association between infections and cognitive ability remained significant after adjusting for other factors known to influence a decrease of general cognitive ability, such as gestational age, birth weight [35], and having more older siblings (i.e. being later in a sibship), which often correlate with socio-economic gradients [36]. However, in this register-based study we might not have been able to adequately control for the residual confounding from other unmeasured factors, such as smoking, alcohol abuse, duration of breastfeeding [37], prenatal nutritional factors [38–40], other rearing circumstances or disease-specific factors which might also correlate with hospital contacts for infections as well as general cognitive ability. Nevertheless, the findings remained significant also after adjustment for an individual or family history of psychiatric disorders and substance abuse which could be associated with several of these unmeasured risk factors as well as socio-economic factors. Furthermore, the possible effect of maternal smoking during pregnancy should to some degree be captured by the included birth weight and gestational age in the adjustments. Moreover, there may be diseases that are associated with both increased susceptibility to infections and somewhat lower cognition (either the disease itself or its treatment). One obvious candidate for such a disease is childhood cancer, which may require treatments that can have an effect on cognitive development and increased risk of infections; however, exclusion of individuals with a prior cancer diagnosis before conscription did not influence the results.

The observed association between parental infection outside the pregnancy period and decreased general cognitive ability in the offspring has not been described before in large-scale studies.

However, the association between infections in the individual and subsequent decreased cognitive ability was only slightly affected by parental exposure to infections that did not explain the associations. Parental infections could also be used as a marker of familial propensity to infection or social factors, which could explain the association with decreased levels of cognitive ability in the child. The association between lower cognitive ability in individuals with parents who had been diagnosed with infection, could also stem from parental infections having been passed on to the child or to maternal infection during pregnancy. Interestingly, maternal infection during pregnancy were associated with the largest decline in cognitive ability in the basic adjustment model, but not when mutually adjusting for parental infections in general. Animal studies using models of prenatal immune activation have suggested possible neurodevelopmental consequences [41–44], and studies of fetal exposure to serologically determined influenza infection in cases with psychosis were associated with poorer cognitive performance; however, not among the control children at presumed low genetic risk for psychosis [45].

Major strengths of the study are the large study sample and that we were able to include all hospital contacts with infections. Furthermore, we were able to adjust for potential confounding variables, including parental education. We only included hospital contacts for infections and the less severe cases of infections not requiring a hospital contact could not be included, which is a limitation on the generalization of the data to less severe infections. However, it is also a strength regarding the actual diseases that have been severe enough to require a hospital contact. Another advantage is that the general cognitive ability was measured in adulthood and that it highly correlates with IQ [27], since IQ scores taken in adulthood tend to remain stable throughout adulthood.

Major limitations are that our sample consisted of males only and those with certain health conditions are exempt from conscription, thus individuals with some disorders (such as intellectual disability) were under-represented in our sample. Hence, we might underestimate the associations between infections and cognitive ability, since individuals with, for instance, encephalitis during childhood and subsequent intellectual disability, would not be included in the study. We made extensive adjustments but nevertheless we can not rule out that social factors, for instance, still have an impact on the results. Additionally, we only had one composite measure of general cognitive ability measured at one time point. The major findings of the study are that recent infections are important to cognition; however, we could not address recovery and the potential clinical importance of a recent infection.

Hospital contacts with infections had occurred in 35% of the individuals prior to conscription and are a common exposure in the population, but the overall observed effect sizes on the general cognitive ability were rather small (0.12 SD). However, the associations with the number, severity and the temporal proximity of the infection displayed larger effect sizes, suggesting that at-risk populations could be identified (with effect sizes up to 0.63 SD). In comparison, the effect sizes of parental education level that have in previous studies been the variable mostly associated with the general cognitive ability displayed effect sizes of up to 1.0 SD in crude models and 0.60 SD in adjusted models [16]. However, the explained variance (R^2^ from the linear regression) for infection was in the crude model only 0.01, whereas the explained variance for parental education was 0.13 and was the variable mostly associated with cognitive ability. In the fully adjusted model the explained variance was 0.14 for the included variables. Nonetheless, at the individual level, these differences could potentially impact in subtle ways on educational outcomes in exposed individuals. Furthermore, from the public health perspective, these otherwise small differences could potentially translate to a shift in educational and clinical outcomes that are more important at a population level [46].

In conclusion, hospital contacts for infections are associated with a decreased general cognitive ability in a dose-response relationship and also with the temporal proximity of the last infection. The cognitive ability might be directly affected by infections or related immune responses; however, heritable and environmental factors associated with infections might also influence the associations.

## Supporting Information

S1 Table ICD-8 and ICD-10 codes for site and type of the infection. (DOC)
