# Supplementary material for: The Association between Infections and General Cognitive Ability in Young Men – A Nationwide Study
Source: PLoS One. 2015 May 13;10(5):e0124005. doi: 10.1371/journal.pone.0124005 (PMC4429968; doi:10.1371/journal.pone.0124005)
Supplement: S1 Table — (DOC) [file pone.0124005.s001.doc]

| **S1 Table. ICD-8 and ICD-10 codes for site and type of the infection** | | |
| --- | --- | --- |
| Infection category | ICD-8 Codes | ICD-10 Codes |
| **Site of infection** | | |
| Sepsis infections | 038 | A40, A41 |
| Hepatitis infections | 070 | B15-B19 |
| Gastrointestinal infections | 000-009, 540 | A00-A09, K35 |
| Skin infection | 680-686, 050-057, 110, 111, 035 | L00-L08, B00-B09, A46 |
| Respiratory infections | 460-486 | J00-J18 |
| Urological infections | 580, 590, 59500, 59501 | N00, N10, N300, N390 |
| Genital infection | 612, 620, 622 | N518B, N70, N71, N72, N76, N770D, N771B, N771L |
| Otitis media infection | 381-382 | H65-67 |
| CNS infections | 04000-04399, 013, 320, 322, 392, 474, 04509-04699, 32300, 07199, 07202, 07501, 07929, 09049, 05201, 05302, 05403, 05501, 05601, 03609, 02701, 06209-06599, 09490-09499 | I02, G00-G07, A17, A80-89, B003, B004, B010, B011, B020, B021, B050, B051, B060, B261, B262, B375, B451, B582, B602, A022C, A548A, A548D, A521A, A521B, A229C, A321, A504, A390, E236A |
| **Type of infection** | | |
| Bacterial infection | 00009-00599,00809-00839,01099-01299, 013, 094, 01400-01899, 02009-02700,02701, 02708,03599, 03609,03610, 03999, 07399, 07699, 07984, 07939, 08899, 08900, 09049, 28940, 32009-32080, 322, 36202, 36600, 36700, 36800, 36805, 36900, 36901,  08099-08399, 09009, 09039, 09059-09399, 09500-09799, 09800-09929, 10009-10499, 36100-36101, 36108-36109, 10009-10499, 38000-38001, 38209-38299, 38309-38399, 39099-39199, 392, 42100-42109,  46100-46109, 10009-10499, 42000, 46201, 46300, 46403, 50199, 50300, 50302, 51000, 51008, 51009, 48101-48308, 50800-50803, 51300-51309, 52259, 52649, 52722, 52838, 52839,52903, 54001, 56200-56219,  56600-56702, 56708, 57703, 59700, 59703, 59000, 59901, 59906, 59009-59099,  59500-59502, 60100, 60102, 60400, 60401, 60739, 61101, 61100, 61200-61499,  61600-61603, 62009-62099, 62200-62219,  63000-63139, 63500-63599, 62949, 64000, 64002, 64009, 64010, 64012, 64020, 64029, 64090, 64092, 64100, 64102, 64110, 64112, 64120, 64122, 64130, 64132, 64140, 64142, 64150, 64152, 64160, 64162, 64170, 64172, 64190, 64192, 64209, 64229, 64300, 64302, 64380, 64382, 64390, 64392, 64490, 64492, 64500, 64502, 64510, 64512, 64520, 64522, 64530, 64532, 64540, 64542, 64550, 64552, 64560, 64562, 64570, 64572, 64580, 64582, 64590, 64592, 67000-67009, 68009-68099,  68108-68299, 68408-68409, 67801, 68501, 68399, 72031, 73299, 76109, 76309,  68600-68608, 71000-71009, 72000-72029 | A022C, A03-A05, A15-A22, A229, A229A, A229B, A229C, A229D, A23-A58, A65-A69, A7, B088D,E060A, E236A, D733,E321, G00, G02 G042, G042A, G050, G060-G062, G079, H000, H030, H043, H440, H050-051, H061, H601, H031A, H031C, H031E, H038A, H061A, H131A, H131H, H131L, H131N, H190A, H190C, H192C,H192E, H192G, H192H, H220D, H220F-G, H320E, H620A, H624A, H670A-B, H660-H664, H700,-701, H750C, H940A,  I301A-D, I320A-D, I00-I02, I398D-H, I410A-E, I430C, I520A, J01, J020, J030, J13-J15, J160, J170, J200-J202, J340, J340A,-D, J340I, J340J, J36, J383B-D, J387-G, J390, J391, J851-J853, J86, J860, J869 , J950A, J398A, J40, J399, J409, K046, K102, K040A, K046A, K052A, K102A-D, K102G-H, K112A, K130A, K140A, K209A, K113, K122, K351, K570--K579, K61, K628N, K650N, K67, K630, K670-K673, K810, K810A, K810C, K859A, K930, L00-L03, L030, L030H-J, L031-L039, L040-L089, M00, M010-M013, M015B, M016, M463, M490, M491, M492, M600, M650, M630A-B, M680A-F, M710, M730-731, M725A, M86, M900, M901A, M902A-902E, N10-N12, N136A-E, N151, N160A-B, N160E-F, N200I, N201I, N30, N300-N303, N303A, N308, N308A, N309, N330, N34, N340-341, N342B, N370A, N390, N410-N413, N431, N450, N459, N481-482, N490-N492, N498A, N498B, N499A-C, N510A-C, N512A, N512B, N511, N511E-J, N518C, N61, N70-N76, N760-N768, N768A, N768B, N770B, N740-N744, N764, N980, O030, O040, O070, O075, O080, O23, O035A-B, O045, O045A-B, O088D-F, O411, O411A-D, O753. O753A, O85, O86, O91, O910-911, O980-O982, O986-O989, P231-P236, P36, P360-P370, P38-39, P390, T814, T814A-D, T814F, T814G-J, T793, T802, T826-827,  T835-836, T845-847, T857, T874, T880, Y410, Y419, Z220-Z224, Z228-Z229 |
| Viral infection | 00880-00890, 040-044, 04509-04699,  05009-05200, 05201,05208-05301, 05302, 05303-05402, 05403, 05404-05500, 05501,  05508-05600, 05601, 05608-05799,  06009-06199, 06209-06599, 06709-06899, 07000-07009, 07199, 07200-07201, 07202, 07203-07209, 07409-07500, 07501,  07508-07509, 07809-07919, 07929,  07949-07982, 07983, 07989-07999, 09990, 46099, 46400-46402, 46408-46599,  47099-47309, 47400-47409, 48099, 76129, 76139 | A08, A080-A084, A60, A630, A80-A90, A9, B00, B000-B004, B004A, B005, B060A-C, B007-B009, B01, B010, B011, B011A, B012, B018-019, B02, B020-B023,  B027-028, B030, B03-B05, B050-054, B058, B06, B060, B068, B069, B07, B079, B08, B080, B081-B083,  O084-085, B088, B088A-B088C, B09, B15-B26,  B260-B263, B268, B269, B27, B270-271,B278-279, B30, B300-303, B308, B309, B33, B330-333, B338, B34,  B340-344, B348, B349, G020, G051, H031B, H031D, H031F, H131C, H131J, H131M, H190D, H19, H191B, H192B, H192D, H192I, H192J, H220C, H220E, H320, H320C, H621A-B, H622A-C, H671A, H671B, I400B, I411A, I411B, J00, J04-J06, J10-J12, J050, J171A-D, J203-207, J210, K770A, K770B, K871A, K871B, M015, M015A, M014, N518B, N770D, N771B, N771G, N771L, O353, O984-985, P230, P35, Z225-226 |
| Other types of infection | 00609-00799,00899,00999, 08409, 08419,08799,08990,09991-09999,  11000-11799, 13009,13019,13099, 13100-13609, 32089-32099, 32300, 36000, 38002-38009,38100-38101, 38108-38199,38400-38401, 42001-42009, 42199, 42299, 46200, 46208, 46209,  46301-46309, 46600-46601, 48100,  48309-48699, 50300-50306, 50308, 50309, 52720, 52809, 54000, 57201, 57209, 76149, 99859, 99939, 54008-54099,  68692-68694, 76319-76399 | A06, A060, A061-A066, A068-069, A07, A070-073,  A078-079, A085, A09, A099, A59, A63, A638, A64, A649, B375, B451, B50-60, B99, B64, B649, B85-B89, G02, G021A, G021B, G021C, G028A, G040, G049, G049A, G049B-C, G052A-C, G052E-G, G052H-J, G079D, H100, H102-H105, H108-109, H131O, H162, H162A, H192A, H192F, H441, H441A, H163, H169, H603, H628, H320D, H650-651, H669, H750, H750A-B, H940, H940B-C, I301, I301E, I400, I400A, I411-412, I412A-B, I521C, I300,  I308,-309, I33, J18, J028, J02-J03, J038-039, J172, J173A-C, J178, J20-22, J208-209, J218-219, J229, J32, J329, J350, J37, J370-371, J40-J42, J998B, J998C, K351A, K770C, K770D, K770E, M016C, M631D-F, M632A, P390B, K35, K350, K750, L303, M631C, M645, M651, M711, N160D, O358B, O983, O986-O989, P238-239, P369, P37, P371-375, P378-379, P390-P399, T89, Y4139, Z22 |
